# ﻿Molecular phylogeny of Lichen Tiger Moths (Lepidoptera, Erebidae, Arctiinae, Lithosiini): a contribution toward classifying Western Hemisphere genera

**DOI:** 10.3897/zookeys.1108.80783

**Published:** 2022-06-24

**Authors:** John D. Palting, Wendy Moore

**Affiliations:** 1 Graduate Interdisciplinary Program in Entomology and Insect Science, University of Arizona, Tucson, Arizona, 85721-0036, USA University of Arizona Tucson United States of America; 2 Department of Entomology, University of Arizona, 1140 E. South Campus Dr., Tucson, Arizona, 85721-0036, USA University of Arizona Tucson United States of America

**Keywords:** Acsalina, Cisthenina, Clemensiina, Eudesmiina, Lithosiina, molecular sequence data, new subtribal classification, phylogenetic analysis

## Abstract

This study analyzes molecular sequence data from one mitochondrial (COI) and two nuclear (28S, RPS5) genes to test the monophyly of previously proposed subtribes of the Lithosiini (Erebidae: Arctidinae), including subtribal assignment of all North American genera that occur north of Mexico. After transferring *Gardinia* W.F. Kirby from Lithosiina to Cisthenina, there is strong support for a monophyletic Lithosiina, which includes three originally unplaced Nearctic genera: *Agylla* Walker, *Inopsis* Felder, and *Gnamptonychia* Hampson. The result of this study removes *Clemensia* Packard and *Pronola* Hampson from Cisthenina and places them in subtribe Clemensiina. We synonymize Eudesmiina under Cisthenina. After these changes, the phylogeny shows strong support for the monophyly of Cisthenina, which includes a further three unplaced Nearctic genera: *Gardinia* Kirby, *Bruceia* Neumögen, and *Ptychoglene* Felder. The monophyly of Cisthenina (including *Eudesmia* and *Gardinia*) is supported by two apomorphies found in adults: the apodemes of the second abdominal sternite are long and the anterolateral processes are fused with the rest of the sternite.

## ﻿Introduction

**Figure 1.**
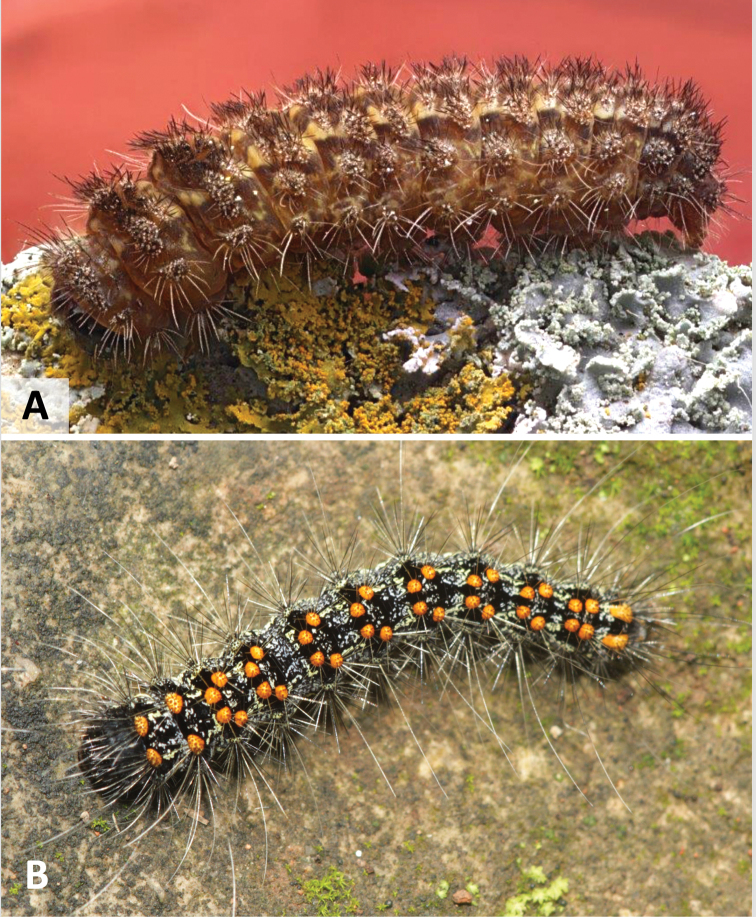
Representative Lithosiini larvae **A***Crambidiamyrlosea* Dyar **B***Inopsismodulata* (Edwards).

**Figure 2.**
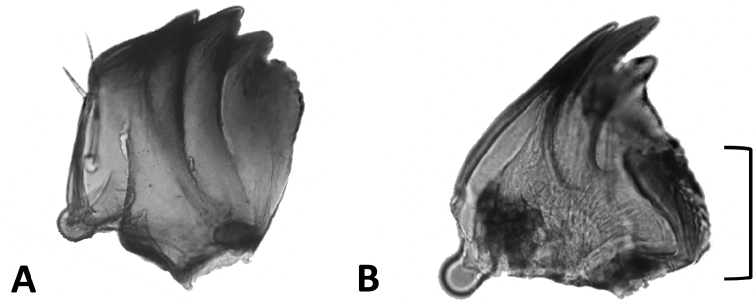
Dissections of the mandible of larvae illustrating two alternate states found among Arctiinae**A** mandible of *Lerinaincarnata* Walker with a blade-like inner margin, as found in Arctiinae tribes other than Lithosiini**B** mandible of *Eudesmiaarida* (Skinner), bracket indicates the mola, an apomorphy of Lithosiini.

Lithosiini (Erebidae: Arctiinae), known as Lichen Tiger Moths, consist of approximately 4000 described species, and have the uncommon ability to feed on lichens (Fig. 1). While other lepidopterans are known to facultatively feed on lichens, only a few groups are known to be obligate lichen feeders. Some authors have suggested most of these are feeding primarily on the algal symbiont of the lichen (Wagner et al. 2008). In the New World, these include members of the Afridini (Nolidae), Elaphriini (Noctuidae) and the Bryophilinae (Noctuidae). Not only do the Lithosiini obligately feed on lichen and algae, they are the only lepidopterans known to sequester phenolics produced by the lichen fungal symbiont (Hesbacher et al. 1995; Wagner et al. 2008; Conner 2009; Scott et al. 2014; Anderson et al. 2017; Scott Chialvo et al. 2018). Lithosiini larvae are secretive, nocturnal, seldom encountered, and poorly known (Wagner 2005; Conner 2009). All Lithosiini larvae that have been examined to date have a mola, a unique flattened, heavily sclerotized area on the inner margin of the mandibles which they use to grind through tough lichen thalli (Fig. 2) (Gardner 1943; Issiki et al. 1965; McCabe 1981; Lafontaine et al. 1982; Rawlins 1984; Garcia-Barros 1985; Habeck 1987; Bendib and Minet 1998; Bendib and Minet 1999; Jacobson and Weller 2002). The ability of the larvae to feed on lichens and sequester associated toxins for their own protection was likely the key innovation that led to the remarkable diversification of this group (Wagner et al. 2008).

**Figure 3.**
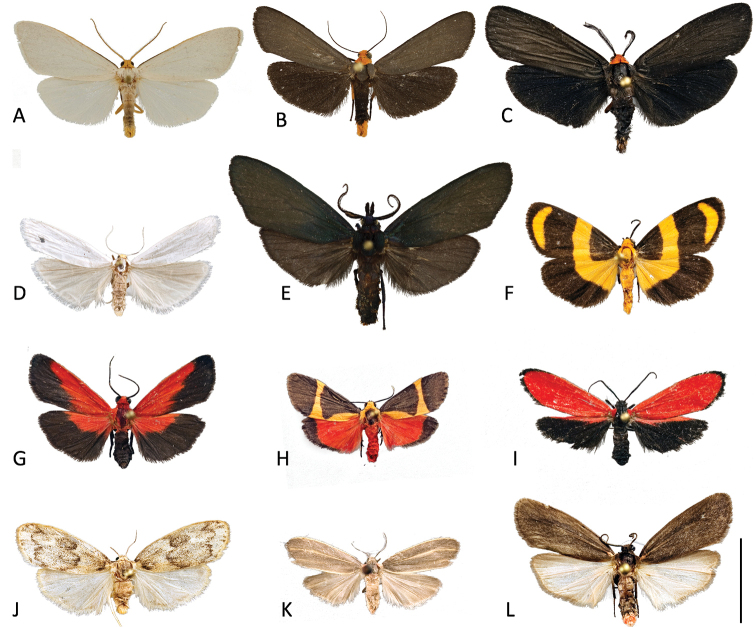
Dorsal views of representative Lithosiini adults from North America **A–D**Lithosiina**E–L**Cisthenina**A***Agyllaseptentrionalis* Barnes & McDunnough **B***Gnamptonychiaventralis* Barnes & Lindsey **C***Inopsismodulata* (Edwards) **D***Crambidiacephalica* (Grote & Robinson) **E***Gardiniaanopla* Hering **F***Eudesmiaarida* (Skinner) **G***Ptychoglenecoccinea* (Edwards) **H***Cisthenetenuifascia* Harvey **I***Lycomorpharegulus* (Grinnell) **J***Bruceiapulverina* Neumögen **K***Haematomisuniformis* Schaus **L***Hypoprepiainculta* Edwards. Scale bar: 1 cm.

Defensive chemicals that the larvae acquire from feeding on lichens are maintained through the pupal stage into the adult (Hesbacher et al. 1995; Anderson et al. 2017; Scott Chialvo et al. 2018). Lithosiini adults are small to medium-sized moths (Fig. 3). Some species have white, gray or brown wing scales and others are brightly and aposematically colored. The audible clicks of some adults warn bats of their distastefulness (Acharya and Fenton 1992). Like their better-studied arctiine relatives, it was suggested that the ability of lithosiines to sequester toxic compounds in the larval stages conveys fitness to the adults (Wagner et al. 2008). Among the arctiines, not only do sequestered toxins provide protection from predators (Eisner and Eisner 1991) and parasites (Singer et al. 2004), they are also critical in pheromone production, courtship success, and can be nuptial gifts that the female passes on to protect her eggs (Conner et al. 1981; Eisner and Meinwald 2003; Jordan et al. 2005). The use of sequestered lichen-derived toxins among members of the Lithosiini remains a wide-open area for research.

Monophyly of the Lithosiini is supported by two larval apomorphies, a mandibular mola (Fig. 2B) and the unique arrangement of labral setae, where M1 is more ventral and far from M2 (Bendib and Minet 1999). In the plesiomorphic condition (non-Lithosiini), M1 and M2 are either in a horizontal line or M1 is slightly dorsad of M2 (Habeck 1987). Lithosiini monophyly is further supported by several molecular phylogenetic studies of the Arctiinae (Zahiri et al. 2012; Zaspel et al. 2014; Zenker et al. 2016).

One lingering question is the classification of the Neotropical genus *Afrida* Möschler which has a confusing taxonomic history. Several authors considered it to belong to Lithosiini (Hampson 1900; Dyar 1913). While the larvae do feed on lichens, they are morphologically distinct, particularly in the shape of their cocoon and that they weave bits of lichen into the structure (Wagner et al. 2011), something no Lithosiini is known to do. Several authors proposed to move this genus from the Erebidae to the family Nolidae, subfamily Afridinae (Holloway 1998; Kitching and Rawlins 1998). More recently, Lafontaine and Schmidt (2010) placed Afridinae as a subfamily of Nolidae, based on COI sequence data and morphology. Although Zahiri et al. (2013a) performed a molecular phylogenetic study of the family Nolidae based on eight gene regions, *Afrida* was not included in their taxon sampling, and thus the phylogenetic placement of this genus has not been tested by molecular-based analysis.

Knowledge of the relationships among the 350 genera classified within the Lithosiini is not well-resolved. Seven lineages within the Lithosiini were either redefined or first proposed by the seminal work of Bendib and Minet (1999). Based on their extensive analysis of morphological characters in adults and larvae (where known) they described and assigned 49 Lithosiini genera to six of these lineages (here considered subtribes), including Cisthenina (26 genera), Eudesmiina (four genera), Acsalina (one genus), Nudariina (15 genera), Endrosiina (two genera) and Phryganopterygiina (one genus). While this work established a baseline and laid the groundwork for future studies of Lithosiini, their taxon sampling was far from complete. They did not include all genera in their classification, and they did not treat the Lithosiina or assign any genera to this group. Jacobson and Weller (2002) included some lithosiines in their pioneering cladistical study of arctiid adult and larval characters, while Scott and Branham (2012) conducted the largest morphology-based phylogenetic analysis of the Lithosiini, including 76 species in 49 genera from each of the proposed seven subtribes. While these studies again supported the monophyly of the Lithosiini as a group, morphology alone failed to elucidate subtribal relationships.

In this study we conduct a DNA-based phylogenetic analysis of the Lithosiini that builds upon three previously published studies (Scott et al. 2014; Zenker et al. 2016; Scott Chialvo et al. 2018), with the aim of including representatives of all genera known from North America north of Mexico (Schmidt and Opler 2008) as well as published sequences from other Western Hemisphere taxa. We propose a new subtribal classification based upon our analyses. The resulting phylogenetic framework and classification provide a baseline for future systematic and behavioral studies of this charismatic group and evolutionary studies of their remarkable defensive chemistry.

## ﻿Materials and methods

### ﻿Gene selection and taxon sampling

Sequences acquired from previous molecular phylogenetic studies of Erebidae (Zahiri et al. 2011; Zahiri et al. 2012; Zahiri et al. 2013b; Scott et al. 2014; Zenker et al. 2016; Scott Chialvo et al. 2018) were downloaded from GenBank and assembled into single gene matrices. Preliminary phylogenetic analyses of the aligned sequences were conducted to determine which gene markers appeared to be most phylogenetically informative and would provide the most complete taxon sampling for our analyses. Based on the results of these preliminary analyses we chose to proceed with one mitochondrial protein-coding gene, cytochrome oxidase I (COI); one nuclear protein-coding gene, ribosomal protein S5 (RPS5); and one nuclear structural gene, the large subunit rRNA D2 loop (28S). Sequences from five species classified in the Erebidae subfamily Aganainae, and representative species of the Arctidinae tribes Arctiniini, Syntomiini, and Amerillini were downloaded from GenBank and included in the single gene matrices as outgroups. Molecular sequence data for 31 additional species, representing 16 genera from the southwestern United States were added. All voucher specimens have been deposited in the University of Arizona Insect Collection (UAIC).

### ﻿DNA extraction, amplification, and sequencing

Total genomic DNA was extracted from the right mesothoracic leg or the abdomen of single specimens using the Qiagen DNeasy Blood and Tissue Kit (Qiagen, Valencia, CA) according to manufacturer suggested protocols. Total genomic DNA was stored in buffer at -80 °C.

Gene fragments were PCR amplified for COI using the primers LCO1490 and HCO2198 (Hebert et al. 2003); the nuclear protein-coding gene RPS5 and nuclear large subunit 28S were amplified using primers and PCR protocols as provided in Scott et al. (2014). PCR products were cleaned, quantified, normalized and sequenced in both directions at the University of Arizona’s Genomic and Technology Core Facility using a 3730 or 3730XL Applied Biosystems automatic sequencer. Chromatograms were assembled into contigs and initial base calls were made for each gene with Phred (Green and Ewing 2002) and Phrap (Green 1999) as orchestrated by Chromaseq ver. 1.5 in Mesquite ver. 3.6 (Maddison and Maddison 2017, 2018). Final base calls were made in Mesquite and ambiguous bases were designated by standard ambiguity codes. GenBank accession numbers for all sequences used in this study are listed in Table 1.

**Table 1.** Sampling of Lithosiini and outgroup species and GenBank accession numbers for sequences used in this study.

	UAIC Specimen Number	RpS5	28S rDNA	COI
Family Nolidae				
*Afridaexegens* Dyar USA: AZ, Cochise Co., Huachuca Mts.	UAIC1148036, UAIC1148037	OM990708	ON006455	ON000160
			ON006456	ON000161
Family Erebidae				
Subfamily Aganainae				
*Asotaheliconia* (Linnaeus)		KC571142	KC570976	KC571044
*Asotaorbona* Vollenhoven		KC571143	KC570977	GWORG305-08
*Neocheradominia* Cramer		KC571144	KC570978	JZAGA909-12
*Peridromeorbicularis* Walker		JN401903		JN401280
Subfamily Arctiinae				
Tribe Amerilini				
*Amerillabrunnea* Hampson		KX300895		KX300223
Tribe Arctiini				
*Cycniatenera* Hübner		KF533651	KF533380	KF533458
*Halysidotatessellaris* J. E. Smith		KF533658		KF533469
*Leucanopsissetosa* Rothschild		KJ723700	KF533400	KJ723706
*Phragmatobiaamurensis* Seitz		KF533679	KF533419	KF533492
*Pygoctenuchaterminalis* Walker Mexico: Sonora, SSW Mesa Tres Rios	UAIC1128849	OM990703	ON006450	
*Virbiacostata* (Stretch) USA: AZ, Pima Co., Santa Catalina Mts.	UAIC1128305	OM990695	ON006437	MF923392
Tribe Syntomiini				
*Amataphegea* (Linnaeus)		HQ006749	KF533352	HQ006238
*Apisacanescens* Walker		HQ006663		HQ006146
*Automolisferrigera* Druce		KF533641		KF533447
**Ingroup**				
Tribe Lithosiini				
Subtribe Acsalina				
*Acsalaanomala* Benjamin		KC571145	KC570980	KJ378646
Subtribe Cisthenina				
*Abrochocisesperanza* Dyar			KC570979	KC571047
*Ardoneatenebrosa* (Walker)		KX361016		KX360798
*Arhabdosia* sp.		KX361034		KX360800
*Balburadorsisigna* Walker			KC570986	KC571053
*Balburaintervenata* Schaus		KX361017	KC570987	KX360802
*Bruceiahubbardi* Dyar USA: AZ, Pima Co., Santa Catalina Mts.	UAIC1128313	OM990689	ON006431	ON000141
*Bruceiapulverina* Neumögen Mexico: Sonora, Sierra del Tigre	UAIC1128312	OM990704	ON006451	KC571055
				ON000157
*Bruceia* sp. 1 Mexico: Sonora, Sierra del Tigre	UAIC1128309	OM990692	ON006434	ON000146
*Bruceia* sp. 2 USA: AZ, Pima Co., Santa Catalina Mts.	UAIC1148030	OM990697	ON006439	ON000144
*Chrysochlorosiamagnifica* Schaus			KC570996	KC571057
*Cistheneangelus* (Dyar) USA: AZ, Pima Co., Tucson Mts.	UAIC1128316		ON006426	ON000136
*Cisthene* sp. USA: AZ, Pima Co., Santa Catalina Mts.	UAIC1148032	OM990690	ON006432	ON000142
*Cisthenemartini* Knowlton USA: AZ, Cochise Co., Huachuca Mts.	UAIC1128318		ON006427	ON000137
*Cisthenekentuckiensis* (Dyar) USA: Texas, Travis Co., Austin	UAIC1148031	OM990698	ON006440	ON000143
*Cisthenetenuifascia* Harvey USA: AZ, Pima Co., Santa Catalina Mts.	UAIC1128319		ON006430	ON000140
*Clemensiamarmorata* (Schaus)		KX300811		KX300245
*Cloesiadigna* Schaus			KC570995	JQ561796
*Cloesia* sp.		KX361038		KX360809
*Dipaenaecontenta* (Walker)		KX361018		KX360815
*Dolichesiafalsimonia* Schaus			KC571000	KC571062
*Eudesmiaarida* (Skinner) Mexico: Sonora, Municipio de Nacori Chico	UAIC1128306	OM990701	ON006448	ON000156
*Eudesmiamenea* (Drury)				MF922663.1
*Euthyonegrisescens* (Schaus)			KC571010	KC571073
*Euthyonepurpurea* (E. D. Jones)		KX361046		KX360823
*Gardiniaanopla* Hering		KC571159	KC571012	KC571075
*Gardiniaanopla* Hering USA: AZ, Pima Co., Santa Catalina Mts.	UAIC1128297		ON006425	ON000135
*Gardiniaparadoxa* Hering		KX361019		KX360825
*Hypermaepha* sp.		KX361049		KX360828
*Hypoprepiacadaverosa* Strecker USA: AZ, Apache Co., Greer	UAIC1148028		ON006446	
*Hypoprepiafucosa* Hübner		KC571162	KC571017	KC571078
*Hypoprepiafucosa* tricolor (Fitch)		KC571163	KC571018	KC571079
*Hypoprepiainculta* Edwards USA: AZ, Cochise Co., Chiricahua Mts.	UAIC1128315	OM990706	ON006453	MH337839
*Hypoprepialampyroides* Palting & Ferguson USA: AZ, Greenlee Co., Blue Ridge Primitive Area	UAIC1128324		ON006441	MH337834
*Hypoprepiaminiate* (Kirby)				MF923793
*Illiceendoxantha* Hampson		KX361050		KX360831
*Lycomorphafulgens* (H. Edwards) USA: AZ, Apache Co., Hannagan Meadow	UAIC1148033		ON006447	
*Lycomorphagrotei* (Packard) USA: AZ, Apache Co., Greer	UAIC1148029	OM990702	ON006449	
*Lycomorpharegulus* (Grinnell) USA: AZ, Greenlee Co., Blue Ridge Primitive Area	UAIC1148034	OM990693	ON006435	ON000147
*Lycomorphodescorrebioides* Schaus			KC571027	KC571088
*Lycomorphodessordida* (Butler)			KC571028	KC571089
*Lycomorphodesstrigosa* (Butler)		KX361051		KX360833
*Metalobosiavarda* (Schaus)		KX361052		KX360836
*Meterythrosiasangala* (H. Druce)			KC571030	KC571030
*Nodozanacf.coresa* Schaus		KX361055		KX360839
*Prepiellasesapina* (Butler)		KX361057		KX360844
*Pronolamagniplaga* Schaus		KX300812		KX300312
*Ptychoglenecoccinea* (H. Edwards)			KC571036	HQ918634
*Ptychoglenephrada* H. Druce		KF533681		KF533497
*Rhabdatomiscoracoroides* Schaus			KC571037	KC571094
*Rhabdatomislaudamia* (H. Druce)	UAIC1128848		ON006429	ON000139
Mexico: Sonora, Sierra La Madera				
*Rhabdatomismandana* (Dyar)		KX361058		KX360845
*Rhabdatomismelinda* (Schaus)			KC571039	KC571096
*Talaracara* Schaus			KC571041	KC571098
*Talaralepida* Schaus			KC571042	KC571099
*Talaranr.mona* Dyar			KC571043	KC571100
*Talarasemiflava* Walker		KX361060		KX360847
Subtribe Endrosina				
*Eugoabipunctata* Walker		JN401906	KF533390	JN401906
*Setinairrorella* (Linnaeus)		KX050605		KX050282
*Stigmatophoramicans* (Bremer & Grey)				KF704470
*Trischalis* sp.				HM906475
Subtribe Lithosiina				
*Agkoniaovifera* Dognin		KX300816		KX300221
*Agyllaargentea* Walker		KX300817		KX300220
*Agyllaargentifera* Walker			KC570981	KC571048
*Agyllaseptentrionalis* Barnes & McDunnough USA: AZ, Cochise Co., Chiricahua Mts.	UAIC1148038	OM990705	4167	ON000158
*Apistosiajudas* Hübner		KX300815		KX300230
*Arevatrigemmis* Hübner		KX300814		KX300233
*Atolmisrubricollis* (Linnaeus)		KC571147	KC570985	ABOLA126-14
*Bruniaantica* (Walker)		HQ006706	KF533366	HQ006193
*Calamidiahirta* Walker		KC571148	KC570990	KC571056
*Crambidiacephalica* (Grote & Robinson) USA: AZ, Navajo Co., Showlow	UAIC1128271	OM990699	ON006442	ON000152
*Crambidiaimpura* Barnes & McDunnough USA: AZ, Gila Co., N. of Winkelman	UAIC1128280	OM990688	ON006428	ON000138
*Crambidiamyrlosea* Dyar Mexico, Sonora, Sierra Alacran	UAIC1148035	OM990696	ON006438	ON000150
*Crambidiapallida* Packard USA: NC, Macon Co, Slick Rock	UAIC1128304	OM990691	ON006433	ON000145
*Crambidiaxanthocorpa* Lewis USA: IN, Tippecanoe Co., Purdue University	UAIC1128323	OM990694	ON006436	ON000148
*Cybosiamesomella* (Linnaeus)			KC570999	ABOLA124-14
*Eilemacomplanum* (Linnaeus) Romania: Torda, Torocko	UAIC1128295		ON006443	ON000153
*Gnamptonychiaflavicollis* (H. Druce)		KC571158	KC571013	KC571076
*Gnamptonychiaventralis* Barnes & Lindsey Mexico: Sonora, Sierra del Tigre	UAIC1128300	OM990707	ON006454	
*Hieragyge* H. Druce		KC571161	KC571015	
*Inopsismodulata* (H. Edwards)		KC571164	KC571020	KC571082
*Lithosiaquadra* (Linnaeus) Bulgaria: Kalimantsi	UAIC1128303		ON006444	ON000154
*Manuleabicolor* (Grote) USA: CO, Gilpin Co., Golden Gate Canyon	UAIC1128293	OM990700	ON006445	ON000155
*Mintopolabraziliensis* Schaus				KX300290
Subtribe Nudariina				
*Asuracervicalis* Walker			KC570983	KC571050
*Barsine* sp.		JN401878	KF533364	JN401286
*Cyanameyricki* Rothschild & Jordan		KC571151	KC570998	KC571061
*Cyana* sp.		JN401876	KF533379	JN401285
*Lyclenepyraula* (Meyrick)		KC571165	KC571022	KC571084
*Lyclenequadrilineata* (Pagenstecher)		KC571172	KC571035	KC571093
*Lyclenereticulata* (C. Felder)		KC571166	KC571023	KC571085
*Lyclene* sp.		KC571168	KC571024	KC571086
*Miltochristaminiata* (Forster)		KC571170	KC571031	KC571090
*Miltochrista* sp.		KC571171	KC571032	KC571091
**unplaced**				
*Heliosiajucunda* Walker		KC571160	KC571014	KC571077

### ﻿Sequence alignment and phylogenetic analyses

Single gene matrices were aligned using default settings in MAFFT v7.474 (Katoh and Standley 2013) and were concatenated in Mesquite. Maximum likelihood analyses were conducted on each gene individually and on the concatenated dataset using IQ-TREE ver. 1.6.10 (Nguyen et al. 2015), as orchestrated by Mesquite. The ModelFinder feature within IQ-TREE (Kalyaanamoorthy et al. 2017) was used to find the optimal character evolution models. The MFP model option was used for 28S, and the TESTMERGE option for the protein-coding genes. The TESTMERGE option sought the optimal partition of sites, beginning with the codon positions in different parts. Analyses of the concatenated data matrix were conducted using the TESTMERGE option, beginning with each codon position for each gene as a separate part (thus, the analysis began allowing for up to 7 parts, three for both of the protein-coding genes and one for 28S). One hundred searches were conducted for the maximum likelihood tree for each matrix. One thousand replicates were used for bootstrap analyses.

## ﻿Results

**Figure 4.**
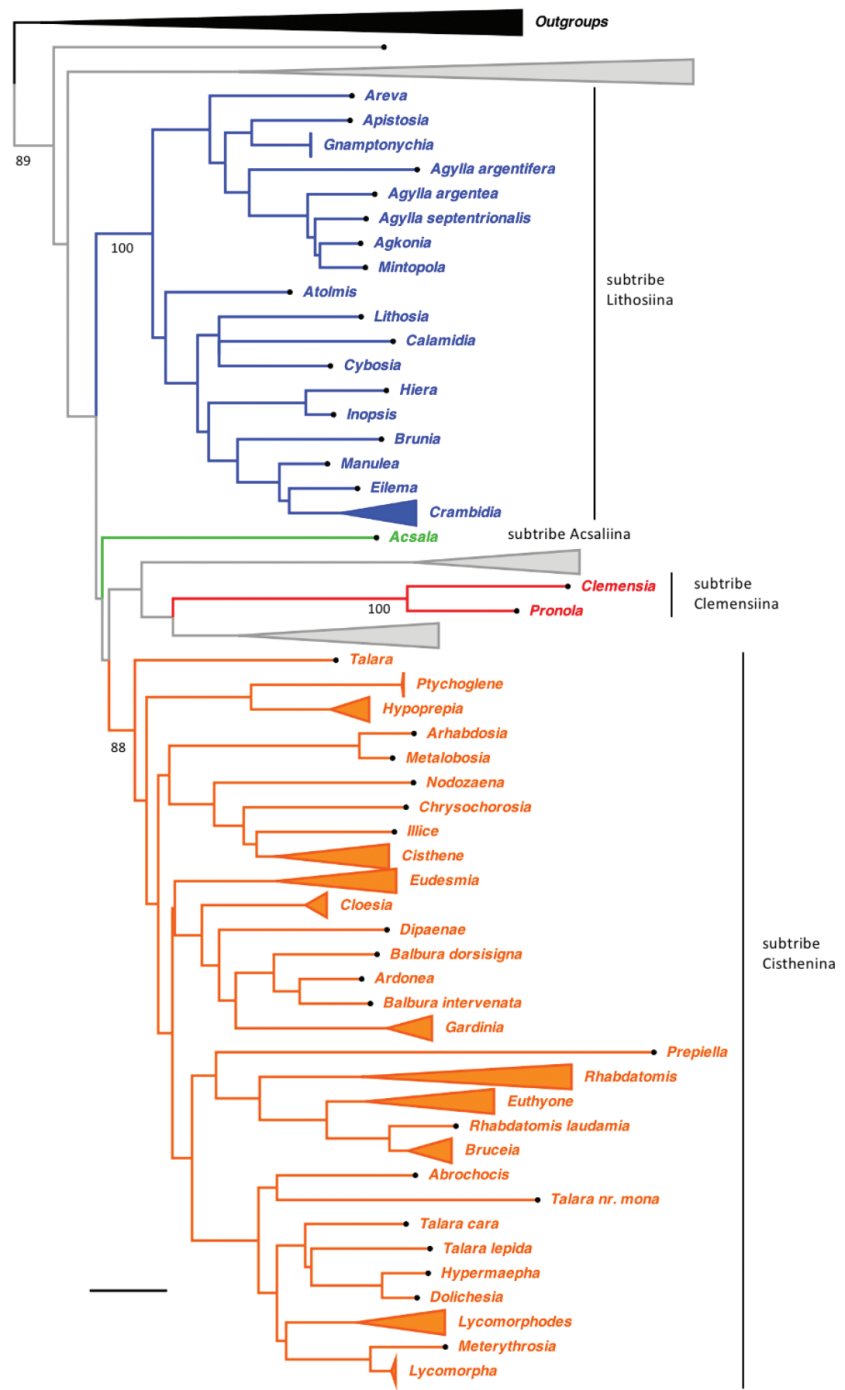
Maximum likelihood tree for the concatenated matrix. Branch lengths are proportional to relative divergence, as estimated by IQ-TREE. Bootstrap values are depicted below branches. Western Hemisphere monophyletic genus-level clades are collapsed and subtribes are colored. Clades that do not include Western Hemisphere species are collapsed and colored gray. See Suppl. material 1: Fig. S1 for the full tree.

A summary diagram of the ML tree for the concatenated dataset is shown in Fig. 4. The full ML tree and all bootstrap values recovered from analyses of the concatenated dataset are shown in Suppl. material 1: Fig. S1. Based on our results we propose several changes to the subtribe classification within Lithosiini as discussed below and summarized in Table 2.

**Table 2.** Proposed classification of Western Hemisphere genera of Lithosiini based on this study with reference to their original placement by Bendib and Minet (1999). Plus symbols (+) indicate that that genus was included in one or more of three molecular-based studies of Lithosiini, including Zenker et al. (2016) (column A), Scott Chialvo et al. (2018) (column B), and this study (column C), and that results support its position in the subtribal classification proposed here. Dashes indicate that that genus was not included in the molecular-based studies. When dashes occur in all three columns, that genus was placed in the proposed subtribal classification by morphology alone.

Proposed subtribal classification	Placement by Bendib and Minet 1999	A	B	C
**Acsalina Bendib and Minet**				
*Acsala* Benjamin	Acsalina	-	-	+
**Cisthenina Bendib and Minet**				
*Abrochocis* Dyar	unplaced	-	-	+
*Ardonea* Walker	unplaced	+	-	+
*Arhabdosia* Dyar	Cisthenina	+	+	+
*Ascaptesyle* Dyar	Cisthenina	+	-	+
*Balbura* Walker	unplaced	+	-	+
*Barsinella* Butler	Cisthenina	-	-	-
*Bruceia* Neumoegen	unplaced	-	+	+
*Callisthenia* Hampson	Cisthenina	-	-	-
*Chrysochlorosia* Hampson	unplaced	-	-	+
*Chrysozana* Hampson	Cisthenina	-	-	-
*Cisthene* Walker	Cisthenina	+	+	+
*Cloesia* Hampson	unplaced	+	-	+
*Dipaenae* Walker	unplaced	+	-	+
*Dolichesia* Schaus	Cisthenina	-	-	+
*Eudesmia* Hübner	Eudesmiina	-	-	+
*Euryptidia* Hampson	Eudesmiina	-	-	+
*Euthyone* Watson	Cisthenina	+	-	+
*Gardinia* Kirby‎	unplaced	+	-	+
*Haematomis* Hampson	unplaced	-	+	-
*Hypermaepha* Hampson	Cisthenina	-	-	-
*Hypoprepia* Hübner	Cisthenina	+	+	+
*llice* Walker	unplaced	+	-	+
*Josiodes* Felder	Eudesmiina	-	-	+
*Leucorhodia* Hampson	Cisthenina	-	-	-
*Lycomorpha* Harris	Cisthenina	-	-	+
*Lycomorphodes* Hampson	Cisthenina	+	-	+
*Maepha* Walker	Cisthenina	-	-	-
*Metallosia* Hampson	Cisthenina	-	-	-
*Metalobosia* Hampson	unplaced	+	-	+
*Meterythrosia* Hampson‎‎	unplaced	-	-	+
*Neotalara* Hampson	Cisthenina	-	-	-
*Neothyone* Hampson	Cisthenina	-	-	-
*Nodozana* H. Druce	unplaced	+	-	+
*Odozona* Walker	Cisthenina	-	-	-
*Paratype* Felder	Eudesmiina	-	-	+
*Prepiella* Schaus	Cisthenina	+	-	+
*Ptychoglene* Felder	unplaced	+	+	+
*Rhabdatomis* Dyar	Cisthenina	+	+	+
*Seripha* Walker	Cisthenina	-	-	-
*Talara* Walker	Cisthenina	+	+	+
**Clemensiina Bendib & Minet**				
*Clemensia* Packard	Cisthenina	+	-	+
*Pronola* Schaus	Cisthenina	+	-	+
**Lithosiina Stephens**	not treated			
*Agylla* Walker	not treated	+	-	+
*Apistosia* Hübner	not treated	+	-	+
*Areva* Walker	not treated	+	-	+
*Atolmis* Hübner	not treated	-	-	+
*Crambidia* Packard	not treated	+	-	+
*Cybosia* Hübner	not treated	-	-	+
*Eilema* Hübner	not treated	-	-	+
*Gnamptonychia* Hampson	not treated	-	-	+
*Hiera* Druce	not treated	-	-	+
*Inopsis* Felder	not treated	-	-	+
*Lithosia* Fabricius	not treated	-	-	+
*Manulea* Wallengren	not treated	-	+	+
*Mintopola* Hampson	not treated	+	-	+

## ﻿Discussion

*Afridaexegens* Dyar was initially included in the taxon sampling to test its potential placement within the Lithosiini or as an outgroup in this analysis. Including it caused long branch attraction, so the ssequences were removed from the matrices. GenBank BLAST searches of 28S, RPS5 and COI all confirm that *Afrida*, long considered by some an arctiine based on hindwing venation, does not belong to Erebidae, supporting the conclusions of Kitching and Rawlins (1998), Holloway (1998), Lafontaine and Schmidt (2010) and Zahiri et al. (2013a), who regarded the Afridinae as a subfamily of the family Nolidae.

### ﻿Monophyly of Lithosiini

Lithosiini monophyly is supported by two morphological apomorphies found in the larvae. Both the unique arrangement of labral setae M1 and M2 and the mandibular mola were present in all Lithosiini larvae reared as part of this study, many of which were previously unknown, including *Agyllaseptentrionalis* Barnes & McDunnough, *Cisthenekentuckiensis* (Dyar), *Gardiniaanopla* Hering, *Crambidiamyrlosea* Dyar, *Eudesmiaarida* (Skinner), *Hypoprepialampyroides* Palting & Ferguson, *Inopsismodulata* (Edwards) and *Lycomorphafulgens* (Edwards).

### ﻿Subtribe Acsalina

*Acsalaanomala* Benjamin occurs on a long branch by itself, supporting the placement of this species in a monotypic subtribe Acsalina. This enigmatic species was placed among the Lymantriidae, however following description of the larval stages feeding on lichens and the presence of a mandibular mola (Lafontaine et al. 1982) it was considered Lithosiini. Bendib and Minet (1999) list many unique apomorphies of the Acsalina, including flightless females, translucent wing vestiture, compound eyes with interommatidial setae, and a primitive hindwing ground plan not found among other Lithosiini. From a biogeographic perspective it is interesting that the Acsalina does not seem to be recently derived from any other temperate lineage contrary to virtually all other Lepidoptera endemic to the Arctic.

### ﻿Subtribe Cisthenina (includes *Gardinia* and Eudesmiina)

When Bendib and Minet (1999) erected the tribe Cisthenini they divided it into the Cistheniti (containing *Cisthene* Walker, *Clemensia* Packard, *Hypoprepia* Hübner, *Lycomorpha* Harris, *Lycomorphodes* Hampson, and *Rhabdatomis* Dyar) and Clemensiiti (containing *Clemensia* Packard, *Pronola* Schaus, *Siccia* Walker, *Hyposiccia* Hampson and *Parasiccia* Hampson). They noted Cistheniti have an unusual resting posture with the antennae facing forward, while Clemensiiti exhibit the plesiomorphic folding backwards of the antennae. They also noted that Clemensiiti rested with the wings flattened rather than roof-like over their back as in Cistheniti.

We find strong support to remove *Clemensia* and *Pronola* (as discussed below) and include thirteen Neotropical genera that were unplaced by Bendib and Minet (1999). These include nine genera (*Balbura* Walker, *Cloesia* Hampson, *Dipaenae* Walker, *Dolichesia* Schaus, *Ilice* Walker, *Metalobosia* Hampson, *Nodozona* Druce, *Ptychoglene* Felder, and *Talara* Walker) which were found to be cisthenines in previous studies molecular-based studies (Zenker et al. 2016; Scott Chialvo et al. 2018) as well as four genera (*Abrochocis* Dyar, *Bruceia* Neumögen, *Chrysochlorosia* Hampson, *Meterythrosia* Hampson) which we include in a molecular-based study and classify for the first time (Table 2). We were not able to obtain fresh specimens of *Haematomis* Hampson for inclusion in our phylogeny, however we speculate based on its small size and resting posture that *Haematomis* belongs to Cisthenina. The wing pattern, particularly the distinctive pink basal wing markings, are consistent with other cisthenines that are thought to be Mullerian mimics of lampyrid beetles, especially some *Hypoprepia* species (*H.lampyroides* and *H.inculta*, for example).

Contrary to previous classifications, this study finds support to include two genera, *Eudesmia* and *Gardinia*, within Cisthenina. Bendib and Minet (1999) classified *Eudesmia* in subtribe Eudesmiina along with three other Western Hemisphere genera: *Euryptidia* Hampson, *Josiodes* Felder, and *Paratype* Felder. This is the first molecular-based phylogenetic study to include a member of the subtribe Eudesmiina. Results indicate that recognizing Eudesmiina as a valid subtribe would render Cisthenina polyphyletic. Therefore, as first revisers we place Eudesmiina in synonymy under Cisthenina, with an expanded concept of the latter. Both names, Cisthenina and Eudesmiina, were published at the same time (Bendib and Minet 1999), so neither name has priority (ICZN Article 24.2). We choose Cisthenina since it is a much more diverse lineage. Given the apomorphies presented by Bendib and Minet for uniting the four genera they placed within the Eudesmiini, we tentatively place them all within Cisthenina (Table 2).

While *Gardinia* was unplaced by Bendib and Minet (1999) it was treated as a member of Lithosiina by several authors (Scott and Branham 2012; Scott et al. 2014). *Gardinia* is a Neotropical genus containing five species, including one species from southeastern Arizona, *Gardiniaanopla* Hering (Fig. 1E). *Gardiniaanopla* is the largest lichen moth in North America with an average forewing length of 25 mm, making it more than twice as large as other Cisthenina (typically with forewing lengths of 10 mm or less). When captured, adults of this nocturnal species produce audible clicks. Among Cisthenina, adults of *Cisthenemartini* are known to produce clicks in response to bat echolocation and are generally avoided by bats (Dowdy and Conner 2016). The clicks of *Gardinia* might be used similarly to warn bats of their distastefulness.

**Figure 5.**
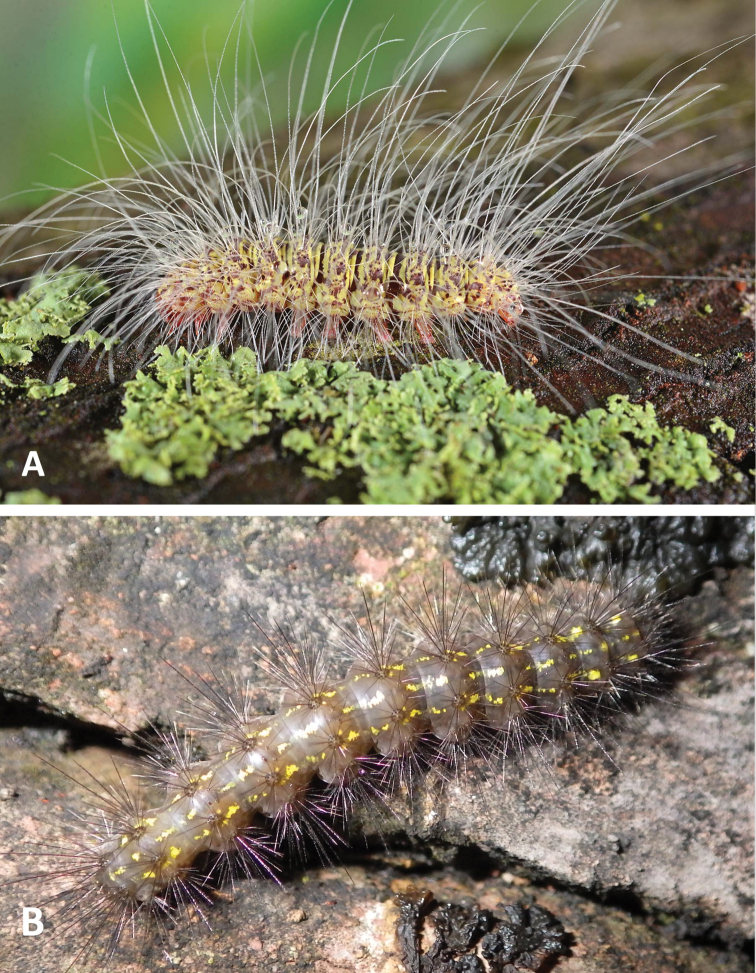
Representative Cisthenina larvae **A***Eudesmiaarida* (Skinner) **B***Gardiniaanopla* Hering.

Cisthenina larvae generally have short, sparse setae and they lack verrucae, which was proposed as an apomorphy for the subtribe (Bendib and Minet 1999). However, the larvae of *Gardinia* (Fig. 5A) and of *Eudesmia* (Fig. 5B), reared as part of this study, possess verrucae making species in these genera the only members of Cisthenina known to have them. *Eudesmiaarida* (Skinner) larvae possess exceedingly long, soft setae, unlike the short, stiff setae characteristic of other Cisthenina (Fig. 5B).

**Figure 6.**
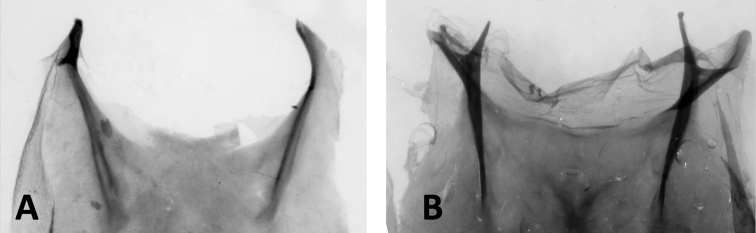
Dissections of the second abdominal sternites of adults illustrating two alternate states found among subtribes of Lithosiini**A** apodemes of *Pygarctiaroseicapitis* (Neumoegen & Dyar) are relatively short and the anterolateral processes articulate with the sternal plate as found in most members of Lithosiina (other than *Agylla*) and in Arctiini**B** apodemes of *Gardiniaanopla* Hering are relatively long and the anterolateral processes are fused with the rest of the sternum as found in members of the Cisthenina, including *Eudesmia* and *Gardinia*.

All members of the *Cisthenina*, as defined here, are endemic to the Western Hemisphere. Among Cisthenina adults, apodemes on the second abdominal sternite are long and the anterolateral processes are fused with the rest of the sternum (Fig. 6). We find that these character states are present in *Eudesmia* and *Gardinia* (Fig. 6) which hold as a strong apomorphies of Cisthenina as redefined in this study.

### ﻿Subtribe Clemensiina Bendib & Minet

**Type-genus**: *Clemensia* Packard.

When Bendib and Minet (1999) erected the tribe Cisthenini they divided it into the Cistheniti and Clemensiiti, with the latter housing *Clemensia*, *Pronola*, *Siccia*, *Hyposiccia* and *Parasiccia*. In addition to the differences they noted in resting posture between the subtribes, they noted three apomorphies of the Clemensiiti, including the presence of a pair of metascutal membranous areas, sternite A2 possessing curved movable anterolateral processes and the presence of a corethrogyne in females.

In this study *Clemensia* falls outside Cisthenina and it forms a highly supported clade with the small neotropical genus *Pronola* Schaus (5 species), the adults of which are similarly sized and have a similar peculiar rounded wing shape. Zenker et al. (2016) found *Clemensia + Pronola* were the sister group of the Oriental genus *Garudinia* Moore. Additional taxon sampling from around the world will be needed to determine the extent of this clade, with the genera *Sicia*, *Hyposiccia* and *Parasiccia* from the Western Hemisphere likely to be included within it. Future research on this clade is likely to be fruitful. Not only is a larger molecular and morphological analysis required, the limited information available on the immatures of species in this clade (McCabe 1981) suggests they are strictly algivores, refusing to feed on lichen at all. This observation, combined with their somber coloring, might indicate they do not sequester lichen phenolics for protection as do all other lithosines.

### ﻿Subtribe Lithosiina

Bendib and Minet (1999) did not treat or assign genera to the Lithosiina. Results of this study indicate the following 13 genera are included in this well-supported clade: *Agylla* Walker, *Apistosia* Hübner, *Areva* Walker, *Atolmis* Hübner, *Crambidia* Packard, *Cybosia* Hübner, *Eilema* Hübner, *Gnamtonychia* Hampson, *Hiera* Druce, *Inopsis* Felder, *Lithosia* Fabricius, *Manulea* Wallengren, *Mintopola* Hampson.

This is the first study to include specimens of *Gnamtonychia* Hampson and *Inopsis* Felder in a molecular-based phylogenetic analysis. Including them in *Lithosiina* is also supported by the shape of the second abdominal sternite (Fig. 6A) and their resting posture with their wings held somewhat flattened and rolled around their abdomen, two traits typical of Lithosiina. *Gnamtonychiaventralis* Barnes and Lindsey occurs in southeastern Arizona and New Mexico and *Inopsismodulata* Edwards occurs in Mexico and is rarely found in southeastern Arizona. These two species are remarkably similar in external appearance as adults, however side-by-side *I.modulata* is a slightly smaller moth with shorter, more rounded wings than *G.ventralis* (Fig. 3B, C). Both species are evidently part of a Mullerian mimicry complex that includes the arctiine *Pygotenuchaterminalis* (Walker) (included here as an outgroup), which is similarly colored and a toxic milkweed-feeder in the larval stages. The larvae of *G.ventralis* are unknown. The larvae of *I.modulata* have distinctive orange to red verrucae and a dark bodies (Fig. 5B), making them conspicuous as they feed on lichens growing on tree branches.

In agreement with Zenker et al. (2016), the molecular phylogeny in this study places *Agylla* within Lithosiina. *Agylla* represents the single largest radiation among Western Hemisphere Lithosiini, with 101 described species found in the Neotropics. Primarily an Old World group, Zenker et al. (2016) proposed the Lithosiina colonized South America from the Holarctic in one or more events. In fact, results presented here likely confirm that there have been at least three incursions from the Old World to the New World (assuming the group originated in Asia as proposed by Zenker et al. 2016). In our analyses we added *A.septentrionalis* Barnes and McDunnough (Fig. 1A), which is restricted to the mountains of southeastern Arizona. Adults of this species look similar to some European members of the genus *Lithosia* Fabricius such as male *L.quadra* Linnaeus included in our analyses. However, the results of this analysis show that these two genera are not closely related, *Crambidia* is instead closely related to *Manulea+Eilema* (Palaearctic/Oriental), whereas *Agylla* is part of a Neotropical clade. We note that adults of *A.septentrionalis* hold their wings “tent-like” over the body rather than flattened and rolled around their abdomens like most Lithosiina. In addition, the adults possess a Cisthenina-like second abdominal sternite (Fig. 6B). Thus, the placement of *Agylla* within Lithosiina, means that these morphological characteristics are more labile than previously thought.

### ﻿Concluding remarks

With a tribe as large as Lithosiini, it is surprising that a subtribal classification was neglected for so long, yet understandable given their worldwide diversity and confounding variation of morphological characters. Beginning with Bendib and Minet (1999), we started to conceptualize how Lithosiini genera might be related. Some of our placements here, such as *Gardinia* among Cisthenina, show that the appearance of the adults does not belie their phylogenetic relatedness. With the apparent lack of morphological apomorphies identified thus far that support subtribal alliances, molecular techniques provide a useful tool for understanding how their diversity evolved. As additional molecular data are published and made available, their evolutionary relationships will become more apparent and hopefully lead to the discovery of morphological apomorphies in both larvae and adults. Presently the whole life history is known for only a small percentage of species. Thus, we have barely scratched the surface in understanding these remarkable lepidopterans and their unique relationship to their lichen hosts and to each other.

## Supplementary materials

Supplementary material 1 Figure S1 Data type Image. Explanation note Maximum likelihood tree for the concatenated matrix. Branch lengths are proportional to relative divergence, as estimated by IQ-TREE, scale bar indicates 0.05 units. Bootstrap values are depicted below branches. Clades are colored by subtribe. https://binary.pensoft.net/file/706011This dataset is made available under the Open Database License (http://opendatacommons.org/licenses/odbl/1.0/). The Open Database License (ODbL) is a license agreement intended to allow users to freely share, modify, and use this Dataset while maintaining this same freedom for others, provided that the original source and author(s) are credited.John D. Palting, Wendy Moore
