# Supplementary figures and images for: ﻿Molecular phylogeny of Lichen Tiger Moths (Lepidoptera, Erebidae, Arctiinae, Lithosiini): a contribution toward classifying Western Hemisphere genera
Source: Zookeys. 2022 Jun 24;1108:119–39. doi: 10.3897/zookeys.1108.80783 (PMC9848871; doi:10.3897/zookeys.1108.80783)

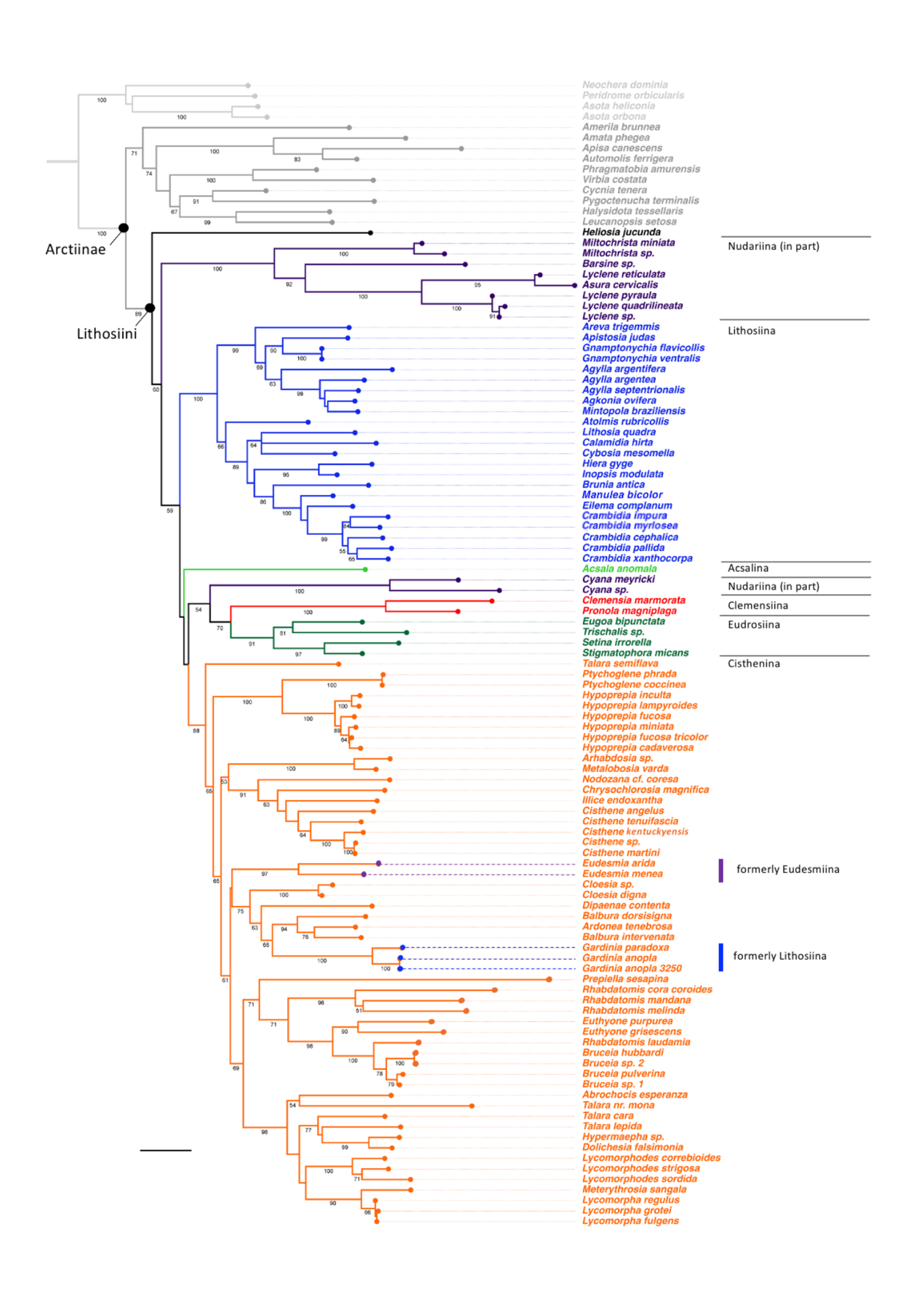

Supplement: Supplementary material 1 — Figure S1 [file zookeys-1108-119_article-80783__-s001.png]
